# Coumarin Content, Morphological Variation, and Molecular Phylogenetics of *Melilotus*

**DOI:** 10.3390/molecules23040810

**Published:** 2018-04-02

**Authors:** Jiyu Zhang, Hongyan Di, Kai Luo, Zulfi Jahufer, Fan Wu, Zhen Duan, Alan Stewart, Zhuanzhuan Yan, Yanrong Wang

**Affiliations:** 1 State Key Laboratory of Grassland Agro-Ecosystems, Key Laboratory of Grassland Livestock Industry Innovation, Ministry of Agriculture, College of Pastoral Agriculture Science and Technology, Lanzhou University, Lanzhou 730020, China; zhangjy@lzu.edu.cn (J.Z.); luok08@lzu.edu.cn (K.L.); wuf15@lzu.edu.cn (F.W.); duanzh12@lzu.edu.cn (Z.D.); yanzhzh16@lzu.edu.cn (Z.Y.); 2 Agricultural Technology Extension and Training Center, Agricultural and Animal Husbandry of Zhongwei, Zhongwei 755000, China; 17711852709@163.com; 3 AgResearch Ltd., Grasslands Research Center, Palmerston North 11008, New Zealand; Zulfi.Jahufer@agresearch.co.nz; 4 PGG Wrightson Seeds, P.O. Box 175, Lincoln, Christchurch 7640, New Zealand; astewart@pggwrightsonseeds.co.nz

**Keywords:** chloroplast sequence, coumarin, *Melilotus*, morphological trait, molecular phylogenetics, nuclear ITS

## Abstract

*Melilotus albus* and *Melilotus officinalis* are widely used in forage production and herbal medicine due to the biological activity of their coumarins, which have many biological and pharmacological activities, including anti-HIV and anti-tumor effects. To comprehensively evaluate *M. albus* and *M. officinalis* coumarin content (Cou), morphological variation, and molecular phylogeny, we examined the Cou, five morphological traits and the molecular characterization based on the *trn*L-F spacer and internal transcribed spacer (ITS) regions of 93 accessions. Significant (*p* < 0.05) variation was observed in the Cou and all five morphological traits in both species. Analysis of population differentiation (Pst) of the phenotypic traits showed that powdery mildew resistance (PMR) had the greatest Pst, meaning that this trait demonstrated the largest genetic differentiation among the accessions. The Pst values of dry matter yield (DMY) and Cou were relatively high. Biplot analysis identified accessions with higher DMY and higher and lower Cou. Analysis of molecular sequence variation identified seven haplotypes of the *trn*L-F spacer and 13 haplotypes of the ITS region. Based on haplotype and sequence analyses, the genetic variation of *M. officinalis* was higher than that of *M. albus.* Additionally, ITS sequence analysis showed that the variation among accessions was larger than that among species across three geographical areas: Asia, Europe, and North America. Similarly, variation among accessions for both the *trn*L-F and ITS sequences were larger than the differences between the geographical areas. Our results indicate that there has been considerable gene flow between the two *Melilotus* species. Our characterization of Cou and the morphological and genetic variations of these two *Melilotus* species may provide useful insights into germplasm improvement to enhance DMY and Cou.

## 1. Introduction

Coumarins, a class of structurally unique and important natural compounds, exist in many plant species in Nature [1,2,3] and present potential medicinal value [4]. Many medicines containing coumarin-based compounds such as warfarin and phenprocoumon are often not specifically linked to coumarin in medical papers [5], and instead, are usually described as having anticoagulant properties or interacting with drugs with anticoagulant properties [4]. The coumarin-derivative prehispanolone from Chinese motherwort (*Leonurus cardiaca*) reduces fibrinogen and blood viscosity and inhibits platelet aggregation [6]. These functions make coumarins important medicinal compounds. Coumarin is a plant secondary metabolite in *Melilotus* [7], and its content varies significantly among different *Melilotus* species, with mean contents ranging from 0.06 to 0.753% of dry matter [8]. *Melilotus* belongs to the family Leguminosae, in the subfamily Papilionoideae, of the clover tribe (Tribus Trifolieae Bronn) [9]. *Melilotus*, commonly referred to as wild alfalfa, consists of 19 species that originated in Eurasia [10]. In China, *Melilotus* is mainly distributed in Liaoning, Shanxi, Gansu and Inner Mongolia [11]. This plant demonstrates good drought, cold and salt tolerance and is used as pharmaceutical material, fodder, green manure, and a soil conservation crop [12]. The most widely-cultivated species, *M. albus* and *M. officinalis* (Figure S1), are primarily used as fodder but also as medicinal plants [13]. The morphology of the two species is very and classification is largely based on flower color: *M. albus* has white flowers, and *M. officinalis* has yellow flowers [14]. *Melilotus* contains various chemical components such as coumarin [15], which possesses high medicinal value, but reliable and accurate methods for differentiating *M. albus* and *M. officinalis* during the vegetative stage are not available. *M. albus* is generally cross-fertile with a low degree of self-fertility in both the annual and biennial types.

Genetic variation in germplasm is the foundation of successful plant breeding, and wild germplasm is a valuable source of new alleles associated with desirable characteristics, such as higher yield, higher or lower coumarin content (Cou), and resistance to pests and diseases. It is essential that plant genetic resources are properly conserved and characterized. There are many examples of the successful use of native or exotic germplasm in plant breeding. To facilitate the use of germplasm resources, basic information on germplasm must be obtained. DNA sequences represent sources of information for biological systematics [16]. DNA sequences are often used to infer phylogenetic trees, which permit visualization of the diversity and relationships in different populations [17,18]. The chloroplast *trn*L-F DNA spacer has higher variability than many other regions in the plant genome [19], and the nuclear ribosomal internal transcribed spacer (ITS) region has alternating gene and spacer regions and tandem repeat structures [20,21,22,23]. A combined cpDNA and ITS approach has been successfully used to analyze systematics in various plants [24,25,26,27]. Sequences of matK, *trn*L-F and ITS were used to evaluate the relationships in Orchidaceae [28,29,30]. Phylogenetic analysis of the Lamiaceae based on sequences of the *trn*L intron, *trn*L-F intergenic spacer, and rps16 intron revealed that Lamioideae was divided into nine tribes [31]. A study combining molecular and morphological data found that *Eremospatha*, *Laccosperma*, and *Oncocalamus* (Palmae) constitute a monophyletic group [32,33]. These three genera share certain morphological characteristics (such as rachis and inflorescences) [34] and possess distinctive traits (including reproductive systems) [32]. Our previous analysis using a chloroplast gene showed that 18 species of the genus *Melilotus* formed a monophyletic clade [35]. Analysis of the genetic diversity and population structure using SSR markers in accessions of the genus *Melilotus* revealed substantial variation among the 18 species and among or within populations [36,37]. Despite the importance of *M. albus* and *M. officinalis* as crops in Eurasia, Africa, and North America, there is a lack of information regarding Cou, genetic variation, and systematics of these plant species. In the present work, a total of 93 accessions, including 42 accessions of *M. albus* and 51 accessions of *M. officinalis*, were collected. To evaluate potential genetic variation and to screen for high dry matter yield (DMY) and higher/lower Cou for future *Melilotus* breeding, we conducted coumarin analysis, morphological evaluation based on five traits and molecular characterization based on the *trn*L-F spacer and ITS region.

## 2. Results

### 2.1. Coumarin Content and Morphological Variation

Analysis of variance indicated significant (*p* < 0.05) genotypic differences among the accessions and within each species for all measured traits (PH, plant height; DMY; LSR leaf:stem ratio; Cou; PMR, powdery mildew resistance; and GH, growth habit) (Table 1). There was a wide range of variation for each of the traits. There were some differences between *M. officinalis* accessions and *M. albus* accessions. The population differentiation (Pst) parameters for all traits varied within a relatively narrow range, from 0.3405 to 0.5251 in *M. officinalis* and 0.3690 to 0.5434 in *M. albus*. The Pst parameter of PMR showed the largest variation (0.5251 in *M. officinalis* and 0.5434 in *M. albus*) among all accessions, and the DMY and Cou values were relatively high (0.4127 and 0.4022 in *M. officinalis* and 0.4642 and 0.4094 in *M. albus,* respectively) compared with the GH value (Table 1).

**Figure 1:**
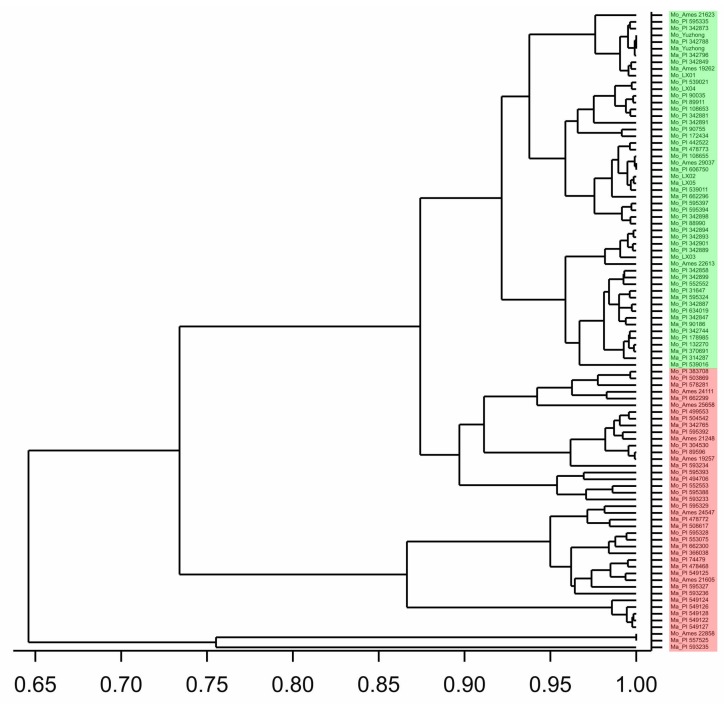
Dendrogram of *M. officinalis* and *M. albus* accessions generated based on six traits using UPGMA as implemented in GenStat software. Two parts were clustered: the green part was largely *M. officinalis* accessions, and the red part was largely *M. albus* accessions. Mo: *M. officinalis*, Ma: *M. albus.*

Cluster analysis of *M. officinalis* and *M. albus* accessions based on six traits was performed using UPGMA, and a dendrogram was inferred (Figure 1). All accessions were divided into two major clusters (green part and red part). The grouping was generated according to species type; most *M. officinalis* accessions grouped together, as did *M. albus* accessions (Figure 1).

**Figure 2:**
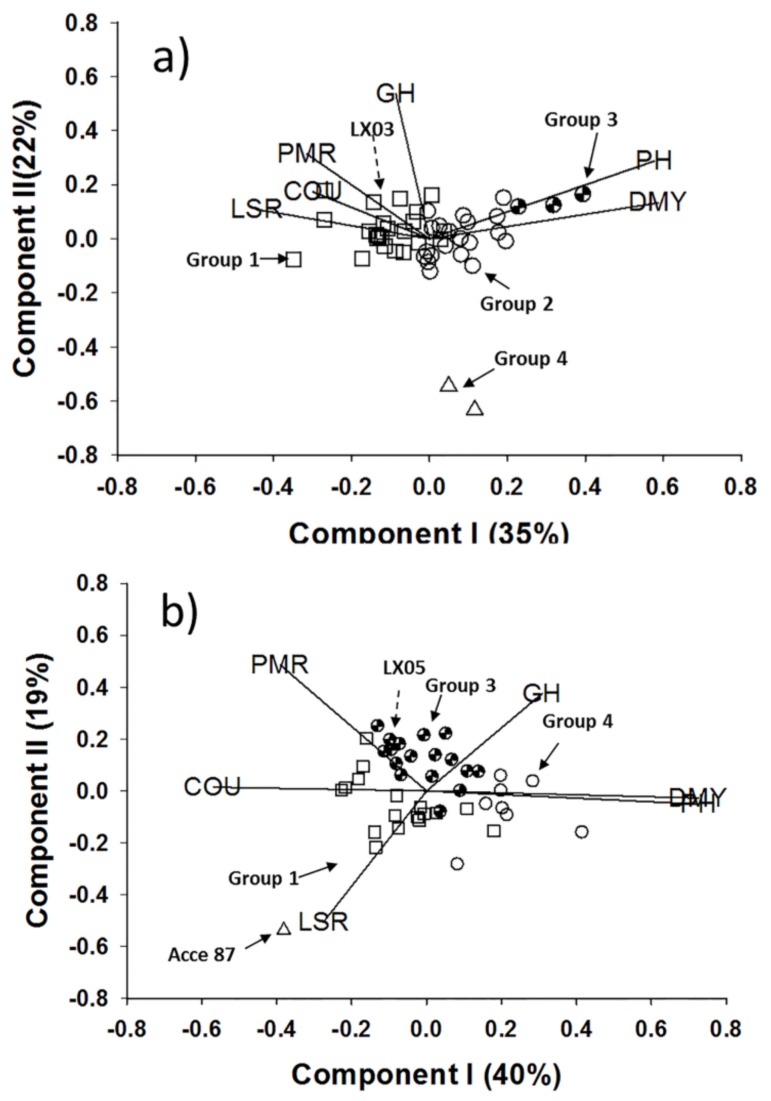
Biplots generated using the accession-by-trait matrix approach for the six traits. PH, plant height; DMY, dry matter yield; LSR, leaf:stem ratio; Cou, coumarin content; and PMR, powdery mildew resistance, measured for (**a**) *M. officinalis* accessions and (**b**) *M. albus* accessions. In each biplot, the variation along components I and II is expressed as a percentage. The different symbols indicate the accession groups generated from the cluster analysis. The two local commercial lines LX03 and LX05 are indicated. The vectors represent the six traits.

Biplots (Figure 2a,b) provided a graphical summary of the multivariate data based on the Cou and five morphological traits measured for all germplasm accessions and checks (commercial cultivars) of *M. officinalis* and *M. albus*. The directional vectors in each biplot, which were generated from the cluster analysis, show the associations among traits and provide a basis to compare accession groups and trait expression. In both *M. officinalis* and *M. albus*, there was a negative association between DMY and Cou. There were a number of germplasm accessions in both species that had higher DMY values than those of the commercial lines LX03 and LX05; this was particularly evident for the accessions in Group 3 for *M. officinalis* and Group 4 for *M. albus* (Figure 2a,b).

The within-group accession means for the six traits measured for *M. officinalis* indicated that Group 1 contained plants with higher Cou and higher LSR values relative to the other groups, whereas Group 3 contained taller plants with higher DMY values and lower Cou (Table 2). For *M. albus*, accession Group 4 contained taller plants with higher DMY values and lower Cou, whereas Acce 87 contained plants with higher Cou and higher LSR values (Table 2).

### 2.2. Alignments and Sequence Variation

Among the 93 *M. albus* and *M. officinalis* accessions, the average lengths of the *trn*L-F spacer and nrITS regions were 459 and 714 bases, respectively; however, the *trn*L-F spacer contains indels. For the aligned *trn*L-F and nrITS sequences, the conserved sites consisted of 452 and 700 bases, respectively, while there were 9 and 14 variable sites, with six and eight parsimony-informative characteristics, respectively (Table S1).

Sequencing of the *trn*L-F spacer in samples of both species from among the 93 accessions identified seven different sequences (Table S2a), with a total of nine variable sites in the *trnL-F* spacer. Three sites were indels, representing five types, and the other six sites were nucleotide substitutions. Total alignment of the ITS regions identified 13 different sequences (Table S2b). The ITS region included 14 variable sites, all of which were substitutions. All *trn*L-F and nrITS haplotype sequences were deposited in the GenBankdatabase under accession numbers KF758417-KF758423 and KF758424-KF758436, respectively.

### 2.3. Phylogenetic Analyses

**Figure 3:**
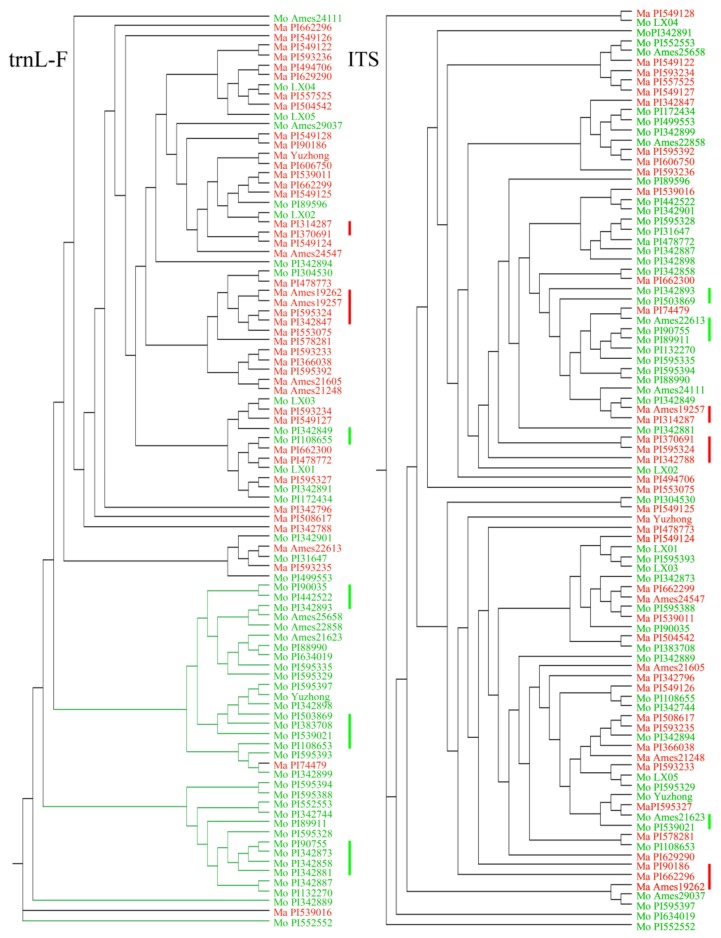
Bayesian tree constructed with MrBayes based on the *trn*L-F and ITS datasets. *M. albus* accessions are indicated with red, *M. officinalis* accessions are indicated with green; the red bars indicate accessions from *M. albus* Group 3 in Table 2, and the green bars indicate accessions from *M. officinalis* Group 1 in Table 2.

Bayesian trees based on the ITS and *trn*L-F sequences are shown in Figure 3. In the ITS tree, the two species were not very well separated. In the *trn*L-F tree based on the 459-base alignment, the accessions tended to separate into two species groups. The combined tree (created in MEGA 6.0) showed greater similarity to the *trn*L-F tree than to the ITS tree (Figure S2). Additionally, the combination of morphological data (Figure 2 and Table 2) and Bayesian trees suggested that some *M. officinalis* accessions (derived from *M. officinalis* Group 1, in Table 2) with high Cou clustered together, while some *M. albus* accessions (derived from *M. albus* Group 3, in Table 2) with relatively high Cou clustered together (Figure 3). A total of seven clusters, marked by green bars, belonged to accessions with high Cou, and five clusters, marked by red bars, belonged to accessions with relatively high Cou (Figure 3). The *trn*L-F tree shared six clusters, and 65% of *M. officinalis* accessions clustered together. Therefore, compared with ITS, *trn*L-F was a better genetic maker to distinguish *M. officinalis* from *M. albus*.

### 2.4. Phylogenetic Relationships of Haplotypes

**Figure 4:**
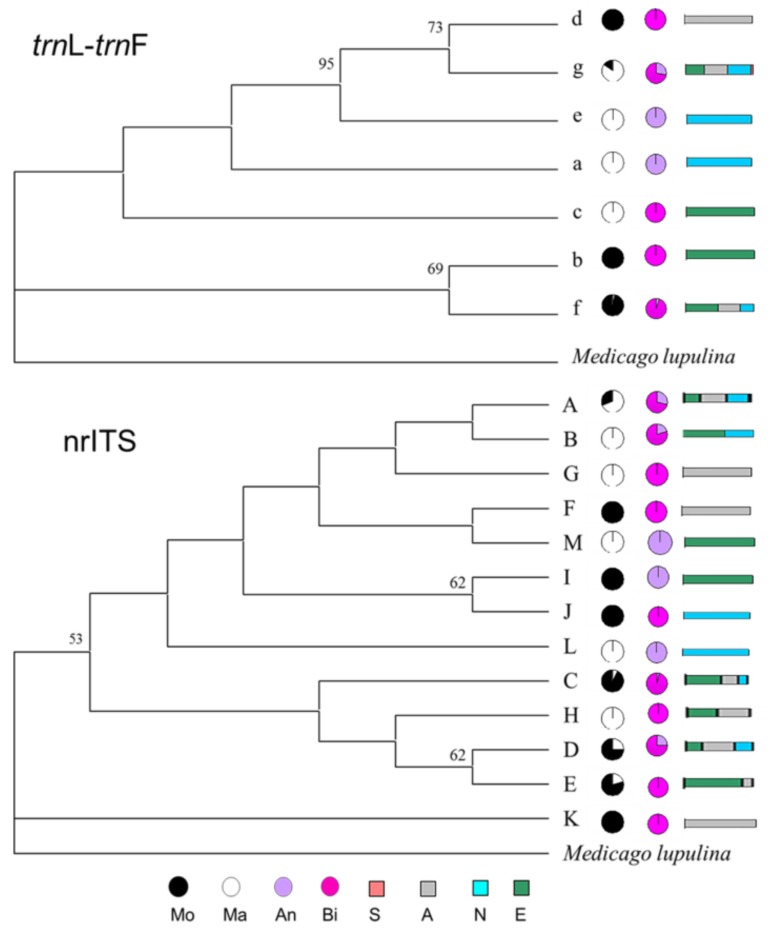
Phylogenetic relationships of haplotypes resolved in *M. albus* and *M. officinalis*. The single maximum parsimonious tree is presented. Bootstrap values (>50%) are denoted above (maximum parsimony). Pie and column charts indicate the frequency of accessions within each haplotype, and unique alleles are indicated by different colors. The first pie chart column shows species distribution, the second column shows growth habit (GH), and the third column shows geographical distribution. *trn*L-F, phylogenetic tree based on cpDNA *trn*L-F haplotypes; nrITS, phylogenetic tree based on nrITS haplotypes (Mo, *M. officinalis*; Ma, *M. albus*; An, Annual; Bi, Biennial; S, South America; N, North America; A, Asia; E, Europe).

The haplotype phylogenetic trees of the *trn*L-F spacer and ITS regions of *Melilotus* are shown in Figure 4, with *Medicago lupulina* as the out group. In the *trn*L-F tree, haplotypes b and f formed a clade: The accessions in haplotype b were all biennial *M. officinalis* that were collected in Europe, and almost all accessions in haplotype f were biennial *M. officinalis* from Asia, Europe, and North America (Figure S3).

Haplotypes a and e were annual *M. albus* that originated from North America, and haplotype c was a biennial *M. albus* from Europe. Haplotypes d and g formed a subclade: haplotype d was a biennial *M. officinalis* derived from Asian stock, and haplotype g comprised annual and biennial *M. officinalis* and *M. albus* from Asia, Europe, and North America. In the ITS tree, haplotype K formed a clade consisting of biennial *M. officinalis* from Asia. Haplotypes B, G, M, L, and H were *M. albus,* and haplotypes F, I, and J were *M. officinalis.* Haplotypes G, F, J, H, and E were biennial, and haplotypes M, I and L were annual.

### 2.5. Genetic Variation within and between Species

The genetic variation of 42 accessions of *M. albus* and 51 accessions of *M. officinalis* were analyzed (Table 3). The aligned *trn*L-F sequence dataset was 459/439 bases in length and contained seven haplotypes: Four *M. albus* and three *M. officinalis*. The ITS region was 714 bases in length and included 13 haplotypes: seven *M. albus*, nine *M. officinalis*, and three shared by the two species. The haplotype variation and nucleotide variation of the two sequences were higher in *M. officinalis* than in *M. albus.*

### 2.6. Molecular Variation within and between Areas

The accessions were divided into four groups according to their origin: Asia, Europe, North America, and South America (Figure S3). AMOVA conducted with the *Melilotus* accessions based on the *trn*L-F spacer showed that most of the variation occurred among species within each geographical area. In contrast, for the nrITS region, most of the variation was present among accessions with low variation among species. AMOVA conducted on three areas (Asia, Europe and North America) showed that the variation was significant among accessions with *trn*L-F and ITS sequences, and only a small amount of variation was present among regions (Figure S3, Table 4).

## 3. Discussion

Coumarins are interesting due to their biological functions, which include antioxidant [38], anti-inflammatory [39], antibacterial [40], and termiticidal properties. These compounds have medicinal value due to their therapeutic properties, including edema reduction and possible anticancer activity [41]. With its variable Cou (ranging from 0 (*M. segetalis* accessions) to 0.943% (*M. indicus* accessions)) [8], *Melilotus* represents a cheap, abundant medicinal plant resource yielding high levels of coumarin. However, the genetic variation within *M. albus* and *M. officinalis* has not been fully investigated prior to this study. Knowledge of the distribution and amount of genetic variation and the systematic relationships among species is important for plant breeding systems utilizing germplasm resources. The initial objective in breeding *Melilotus* is to identify agronomically adapted low/high-coumarin germplasm to be used as initial breeding material. Historically, a number of *Melilotus* varieties were released throughout China, Canada, and the United States (largely before the 1980s). However, distinct varieties are not readily available today due to widespread hybridization among the different varieties, and no new varieties have been released in recent years.

This study indicated significant (*p <* 0.05) differences among accessions in terms of Cou and the morphological traits of PH, DMY, LSR, PMR, and GH. There was also genetic variation between the two species for these traits. Interspecific variation in the two *Melilotus* species (93 accessions with 87 strains) was studied using morphological characteristics. The results showed that *M. albus* and *M. officinalis* had greater variation among accessions. *F_st_* was affected by genetic drift, migration rate, and selection [42]. PMR demonstrated the largest genetic differentiation capacity among the accessions, while DMY and Cou values were relatively high. The potentially higher genetic differentiation capacity of DMY and Cou provides a genetic basis for breeding cultivars with high DMY and high/low Cou. Cluster analysis of the six traits indicated that all *M. albus* accessions or all *M. officinalis* accessions did not cluster into a single group (Figure 1). Thus, hybridization has occurred between *M. albus* and *M. officinalis*.

*M. albus* and *M. officinalis*, which have low to high coumarin levels, have been identified in different accessions and environments [14,43]. A comprehensive investigation of coumarin concentrations in 149 accessions belonging to 15 *Melilotus* species showed that the mean Cou ranged from 0.06 to 0.753% of dry matter [8]. Our study identified a negative correlation between the DMY and Cou in two species. There were a number of germplasm accessions with higher DMY values and lower Cou than the commercial lines LX03 and LX05 or accessions with lower DMY values and higher Cou than these commercial lines. Furthermore, 40 half sib (HS) families obtained from cross-pollinating Group 3 (Table 2) individuals of *M. officinalis* were assessed for their performance across two contrasting locations to identify materials with superior agronomic performance [44]. Any plant improvement program that aims to develop new cultivars of *Melilotus* for agriculture should aim for high DMY and high/low Cou (high Cou for medicine and low Cou for forage). This information is important for breeding programs targeting high/low Cou in *Melilotus*.

Sequence variation studies showed that the *trn*L-F spacer had three indels containing a total of 26 bases representing variable sites. Rarely, indels were detected in *M. officinalis*; this phenomenon may be due to hybridization with *M. albus*. Additionally, the Bayesian tree for the *trn*L-F spacer showed that *M. officinalis* accessions and *M. albus* accessions tended to separate into two species groups. Therefore, the *trn*L-F spacer could be used as a barcode to identify *M. albus* and *M. officinalis* because different indels in the two species effectively clustered them into two groups [44].

The higher sequence variability in the ITS region compared with cpDNA, a phenomenon that has also been observed in many other taxa [45,46], may lead to incongruence in the phylogenetic tree. The genetic variation in *M. officinalis* was higher than that in *M. albus* in both sequences, as revealed by analyzing haplotypes and nucleotide variation (Table 3). The amplification of nine microsatellite DNA loci from *Melilotus* also showed that both the allelic variation and expected heterozygosity were slightly lower for *M. albus* than for *M. officinalis*, with heterozygote deficits at several loci [47]. *M. officinalis* originated in Europe, and *M. albus* originated in Western Asia [48], suggesting that the different geographical and evolutionary histories of these species may also cause differences in their genetic variation. Genetic variation analyses by region showed that genetic variation in the ITS sequence among accessions was higher than that among species in all three regions, but genetic variation in the *trn*L-F sequence among accessions was lower. Analysis of the three regions indicated that the genetic variation among accessions was higher than that among species for both markers. The difference in variation between the two sequences in all three regions was mainly attributable to the indels in the *trn*L-F sequence. Germplasm exchange and introduction throughout the world are very frequent, which may also influence the genetic variation in plant species in different regions. Haplotype phylogenetic analyses showed that the haplotypes of the two species were mixed together in the *trn*L-F and ITS trees. The results indicated that *M. albus* and *M. officinalis* have a close relationship. Karyotype analyses [49] and interspecific phylogenic relationships [35] also showed that *M. albus* and *M. officinalis* are closely related within the *Melilotus* genus. In addition, incomplete lineage sorting [50] and hybridization between, and within, species [51] may cause phylogenetic incongruence. The positions of *M. albus* and *M. officinalis* in the phylogenetic tree indicate that they have shared haplotypes, although they have obvious morphological differences. Therefore, there is a certain amount of gene flow between the two species. It might be argued that interspecific hybridization, a widespread phenomenon that has markedly contributed to variation and speciation in the plant kingdom [52,53,54,55], contributed to the genetic introgression of *M. albus* and *M. officinalis*. In this report, some accessions sharing similar phenotype trait data and Cou clustered together by *trn*L-F and ITS sequences. Therefore, the combination of Cou, morphological variation and molecular phylogenetics identified accessions of *M. albus* and *M. officinalis* based on first-year establishment data for medicinal purposes and low Cou for forage.

## 4. Materials and Methods

### 4.1. Germplasm

A total of 93 accessions were evaluated in this study, comprising 42 accessions of *M. albus* and 51 accessions of *M. officinali*s (Table S3). All seeds of accessions were obtained from the National Plant Germplasm System (NPGS, USA). In addition to the germplasm accessions, two cultivars (LX03, *M. officinalis* and LX05, *M. albus*) obtained from the local seed industry (Tongwei Longzhong Forage Seed Company, Tongwei county, Gansu province, China) were included in our study. The collection contained 36 wild, 15 cultivated, and 12 bred strains, as well as 30 accessions with uncertain pedigree according to the germplasm bank passport information (Table S3).

### 4.2. Field Experiment

#### 4.2.1. Preparation of Plant Material

Sixty seeds from each accession were sown in plastic pots (13 cm × 12 cm) containing a potting mix of sandy soil and peat (1:1 by volume). After germination, the seedlings were planted into pots with the soil mixture in a glass house. Twenty seedlings of each accession, uniform in size, were transplanted into the field after eight weeks. The field trial was established at Yuzhong (35°57′ N, 104°09′ E) in Gansu Province, China, in June 2012. The long-term annual rainfall at Yuzhong is 382 mm, with an evaporation of 1343 mm and an average temperature of 6.7 °C throughout the year.

#### 4.2.2. Field Trial

The field trial had a randomized complete block design with two replicates. Each accession was represented by 10 plants per replicate grown at a plant spacing of 30 cm × 30 cm. Prior to planting, the experimental area was prepared as a seedbed. Post planting, all trial management was conducted according to local procedures, and weeding occurred regularly. No irrigation was applied.

#### 4.2.3. Agronomic Traits

As most *Melilotus* species are biennial, we did not consider first-year establishment data to be important for our breeding program; all measurements were carried out during the second year. Data were collected for the following traits: PH, DMY, LSR, Cou, and PMR. PH (cm) was measured with ten plants per accession per replicate at the early flowering stage, and the aboveground plant parts were harvested. The plant material was air-dried to a constant weight at room temperature and then separated by hand into leaf and stem fractions. The mass composition of whole plants (DMY) was calculated from the weights of the leaves and stems, and the LSR was calculated. The Cou (% of DMY) was measured following the UV method of Tang [56], in which 1.0 g of well-mixed plant material was extracted for 40 min in 50 mL of 50% ethanol using an ultrasonic bath. After ultrasound-assisted extraction, all of the filtrate was combined, and its volume was reduced using a rotating vacuum evaporator. Then, 10 mL of methanol was added, and the Cou was quantified using a UV-VIS spectrophotometer via light absorption at 310 nm, which is the maximum absorption of coumarin residues. The PMR of the plants was scored in the field following the method of Xie [57]. The plants were scored under field conditions during early autumn, which is a period of high powdery mildew infection, using a scale of 0 for no visible symptoms to 4 for a highly susceptible reaction.

#### 4.2.4. Analysis of Variance and Pattern Analysis

To evaluate accession differentiation at the phenotype level, the variance component among accessions divided by the sum of variance components among and within accessions was computed for each trait (Pst). A key objective in the breeding program for both *M. officinalis* and *M. albus* is to improve the DMY and the Cou. Therefore, a combination of cluster analysis and principal component analysis (PCA) [58,59] was conducted to summarize the accession-by-trait data matrix for the traits PH, DMY, LSR, Cou, PMR, and GH. Cluster analysis was conducted by performing UPGMA clustering in GenStat 18th Edition (https://www.vsni.co.uk/webstore/old-webstore/software/ genstat/). Biplots were generated from the PCA to enable the assessment of genotypic variation among germplasm accessions on a multivariate scale. Only traits for which there was significant (*p* < 0.05) genotypic variation among the accessions were included in the pattern analysis.

### 4.3. Molecular Phylogeny

#### 4.3.1. DNA Extraction, Amplification, and Sequencing

Young leaves were collected from twenty plants of each accession, frozen in liquid nitrogen, and stored at −80 °C until they were used for DNA isolation via the SDS extraction method [60]. Preliminary universal primer scanning was conducted on 10 individuals sampled from 10 different accessions. ITS1, 5.8s, and ITS2 were amplified as a single molecule using the flanking primers EC-1 (5′-GAGGAAGGAGAAGTCGTAAC-3′) and EC-2 (5′-GTTCGCTCGCCGTTACTAAG-3′) [61]. The *trn*L-F spacer was amplified using the primer pair *trn*L (CGAAATCGGTAGACGCTACG) and *trn*F (ATTTGAACTGGTGACACGAG) according to Taberlet et al. [62]. PCR amplification [63] was carried out in a 30 μL volume containing 12 μL of deionized water, 15 μL of Takara Taq DNA polymerase master mix, 1 μL of each primer (5 pmol/mL), and 1 μL of template DNA (40 ng/mL). The cycling conditions for amplification consisted of a single cycle at 94 °C for 3 min for initial denaturation; 36 cycles at 94 °C for 30 s, 52 °C for 45 s, and 72 °C for 1 min; and a final extension at 72 °C for 10 min. The PCR products were checked for fragment length and separation in 1% agarose gels and then sequenced using a Big Dye kit with the appropriate primers in an ABI 3730 DNA sequencer at Shanghai Shenggong Biotechnological, Ltd. (Shanghai, China).

All raw sequences were checked by Chromas software (Shanghai Shenggong Biotechnological, Ltd. Shanghai, China), and for sequences that were inaccurate, the corresponding PCR samples were re-prepared and sequenced again. If sequence quality was acceptable, the sequences were aligned by using DNAMAN to ensure their identity as *Melilotus* sequences.

#### 4.3.2. Phylogenetic Analyses

The sequences were aligned in ClustalW (within MEGA 6.0) [64] and manually adjusted using MEGA 6.0 [65]. Maximum parsimony analyses involved a heuristic search strategy with 1000 replicates of random sequence addition in combination with tree bisection and reconnection (TBR) branch swapping in MEGA 6.0. A phylogenetic tree was constructed with ITS and *trn*L-F combined sequences in MEGA 6.0. Separate ITS and *trn*L-F phylogenetic trees were inferred by Bayesian inference via MrBayes-3.2.6 [66] (two runs of 10,000,000 generations with four chains for ITS and three chains for *trn*L-F) under a GTR model with base frequencies, gamma shape parameter, and proportion of invariants estimated from the data (referred to as GTR + I + G). All character states were treated as unordered and equally weighted. Informative insertions and deletions (indels) were coded as binary characters (0, 1) according to Graham et al. (2000). A strict consensus tree was constructed from the most parsimonious trees. Haplotype diversity (*Hd*) and nucleotide diversity (π) analyses were conducted using DnaSP v5 [67]. Fu’s *Fs* test [68] for all sequences was used to test each haplotype group to determine if all samples followed neutral evolution. Inter-accession differentiation between, and within, the two species was evaluated by AMOVA [69] using Arlequin software, version 3 [70]. The sequences of the outgroup *M. lupulina* were downloaded from GenBank (KX167243.1 and KU600399.1). The multiple sequence alignments and phylogenetic trees were submitted to TreeBase (http://purl.org/phylo/treebase/phylows/study/TB2:S22217).

## Figures and Tables

**Table 1:** Ranges, means, and population differentiation (Pst) for traits measured in *M. officinalis* and *M. albus* accessions ^†^.

Trait	*M. Officinalis*				*M. Albus*			
	Range	Mean	Significance	Pst	Range	Mean	Significance	Pst
PH	20–100	62	*	0.4254	11–161	65	*	0.4103
DMY	4–41	17	*	0.4127	0.5–43	16	*	0.4642
LSR	0.7–1.8	1.17	*	0.4238	0.3–3.4	1.00	*	0.4138
Cou	0.3–1.5	0.83	*	0.4022	0.2–1.3	0.73	*	0.4094
PMR	1–4	2.75	*	0.5251	1–4	2.16	*	0.5434
GH	1–2	1.96	*	0.3405	1–2	1.56	*	0.3690

* Significantly different at *p* < 0.05; ^†^ PH, plant height (cm); DMY, dry matter yield (g/plant); LSR, leaf:stem ratio; Cou, coumarin content (%); PMR, powdery mildew resistance (0, 1 to 4 scale); GH, growth habit (1, annual; 2, biennial); Pst, population differentiation.

**Table 2:** Within-group accession means for each of the six traits based on the four clusters generated from the cluster analysis of *M. officinalis* and *M. albus* germplasm entries evaluated in Yuzhong, China.

Species	Groups	No. in Group	PH	DMY	LSR	COU	PMR	GH
*M. officinalis*	Group 1	23	55	12	1.255	0.908	3.39	2
	Group 2	20	43	20	1.104	0.79	2.15	2
	Group 3	3	92	35	1.04	0.526	2.67	2
	Group 4	2	48	17	0.98	0.673	1.5	1
*M. albus*	Group 1	17	58	13	0.974	0.769	2.29	1
	Acce 87	1	11	0.52	3.421	1.158	1	1
	Group 3	17	63	14	0.828	0.771	2.53	2
	Group 4	8	94	29	1.115	0.511	1.25	1.875

PH, plant height (cm); DMY, dry matter yield (g/plant); LSR, leaf:stem ratio; Cou, coumarin content (%); PMR, powdery mildew resistance (0, 1 to 4 scale); GH, growth habit (1, annual; 2, biennial); Acce 87 is the code of accession PI 557525.

**Table 3:** Summary of genetic variation for the 93 accessions of *Melilotus.*

Sequence	Dataset	Length	*N*	*h*	*Hd* ± SD	π ± SD
*trn*L-F	*M. albus*	459	44	4	0.171 ± 0.075	0.0017 ± 0.0015
	*M. officinalis*	439	49	3	0.357 ± 0.064	0.0050 ± 0.0016
	All	459	93	7	0.592 ± 0.021	0.0069 ± 0.0013
ITS	*M. albus*	714	44	7	0.490 ± 0.091	0.0010 ± 0.0009
	*M. officinalis*	714	49	9	0.703 ± 0.645	0.0017 ± 0.0014
	All	714	93	13	0.701 ± 0.036	0.0017 ± 0.0014

*N*, number of sequences; *h*, number of haplotypes; *Hd*, haplotype diversity; *π*, nucleotide diversity; SD, standard deviation.

**Table 4:** Analyses of molecular variance (AMOVAs) for *Melilotus* accessions based on *trn*L-F and nrITS sequences.

Region	Sequence	Source of Variation	d.f.	Sum of Squares	Variance Components	Percentage of Variation	*F_st_*
A	*trn*L-F	Among species	1	3.199	0.206	55.820	0.558
		Among accessions	31	4.737	0.163	44.180	
		Total	32	7.935	0.370		
	ITS	Among species	1	0.549	0.016	4.710	0.047
		Among accessions	31	9.355	0.323	95.290	
		Total	32	9.903	0.339		
E	*trn*L-F	Among species	1	5.417	0.347	75.020	0.750
		Among accessions	34	3.583	0.116	24.980	
		Total	35	9.000	0.463		
	ITS	Among species	1	2.419	0.134	30.320	0.303
		Among accessions	34	10.152	0.308	69.680	
		Total	35	12.571	0.442		
N	*trn*L-F	Among species	1	3.841	0.400	78.650	0.786
		Among accessions	26	2.714	0.109	21.350	
		Total	27	6.556	0.509		
	ITS	Among species	1	0.882	0.065	18.850	0.188
		Among accessions	26	6.733	0.281	81.150	
		Total	27	7.615	0.346		
A & E & N	*trn*L-F	Among regions	3	1.463	0.012	3.850	0.038
		Among accessions	92	23.295	0.268	96.150	
		Total	93	95.000	24.758		
	ITS	Among regions	3	1.855	0.014	3.840	0.038
		Among accessions	92	29.808	0.339	96.160	
		Total	93	31.663	0.352		

A, Asian; E, Europe; N, North America; d.f., degrees of freedom; *F_st_*, Fixation index.

## Supplementary Materials

Supplementary materials can be accessed at: http://www.mdpi.com/1420-3049/23/4/810/s1. Figures S1–S3; Tables S1–S3.
