# Supplementary material for: Coumarin Content, Morphological Variation, and Molecular Phylogenetics of Melilotus
Source: Molecules. 2018 Apr 2;23(4):810. doi: 10.3390/molecules23040810 (PMC6017091; doi:10.3390/molecules23040810)
Supplement: Supplementary file 1 [file molecules-23-00810-s001.zip › Supplementary files/Supplementary Figure Legend.docx]

**Figure S1.** Pictures of fields containing *M. albus* and *M. officinalis*. The left picture shows *M. albus*, and the right picture shows *M. officinalis*.

**Figure S2.** Topology resulting from a neighbor-joining analysis of the combined dataset containing cpDNA genes (*trn*L-F) and nrDNA (ITS) using MEGA 6.0. The accessions belonging to *M. albus* are indicated with red triangles, *M. officinalis* with green dots, and outgroup species with blue squares.

**Figure S3.** Geographical origin of the specimens from South America (S), North America (N), Asia (A), and Europe (E).
