# Supplementary figures and images for: Coumarin Content, Morphological Variation, and Molecular Phylogenetics of Melilotus
Source: Molecules. 2018 Apr 2;23(4):810. doi: 10.3390/molecules23040810 (PMC6017091; doi:10.3390/molecules23040810)

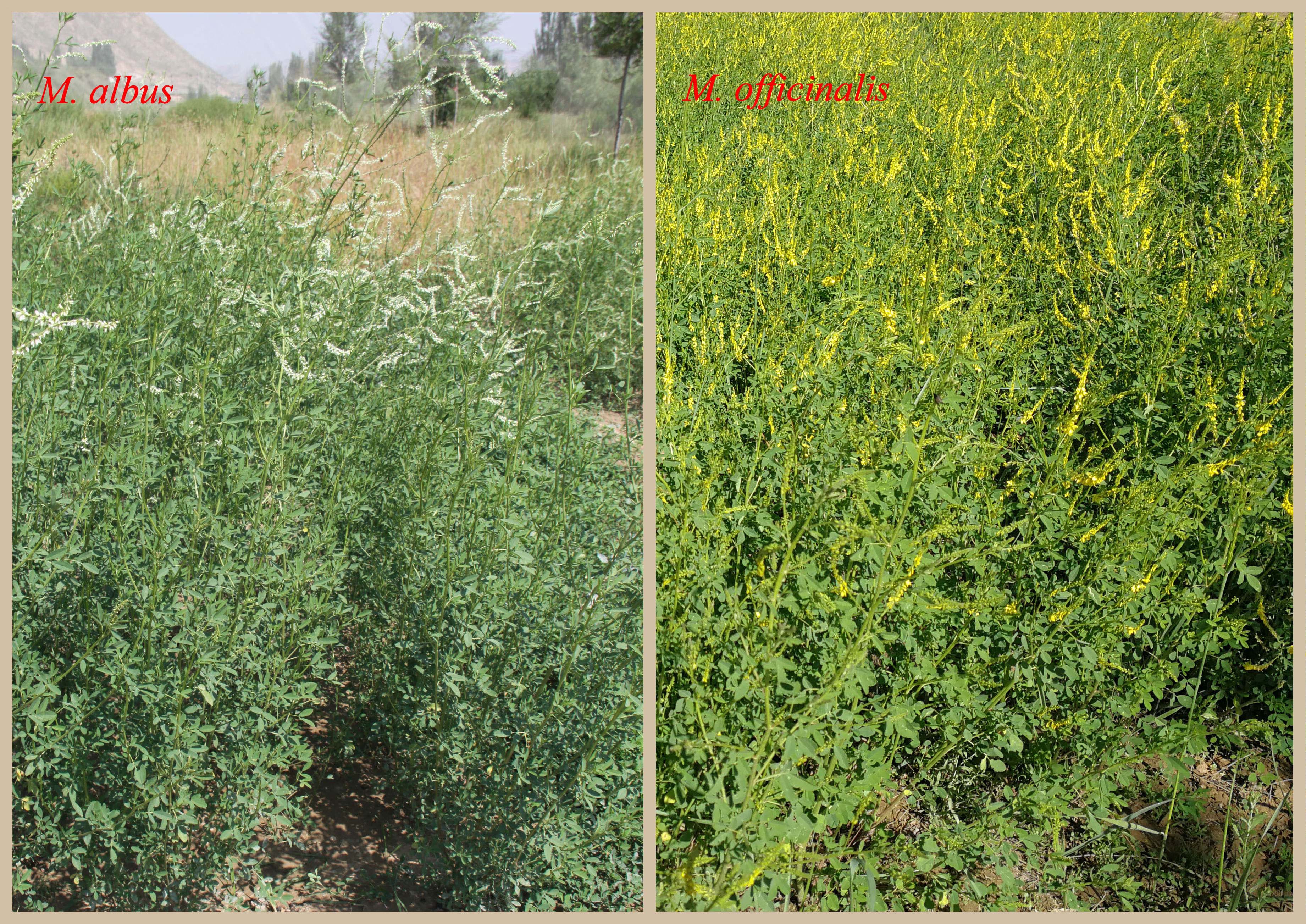

Supplement: Supplementary file 1 [file molecules-23-00810-s001.zip › Supplementary files/Figure S1.tif]

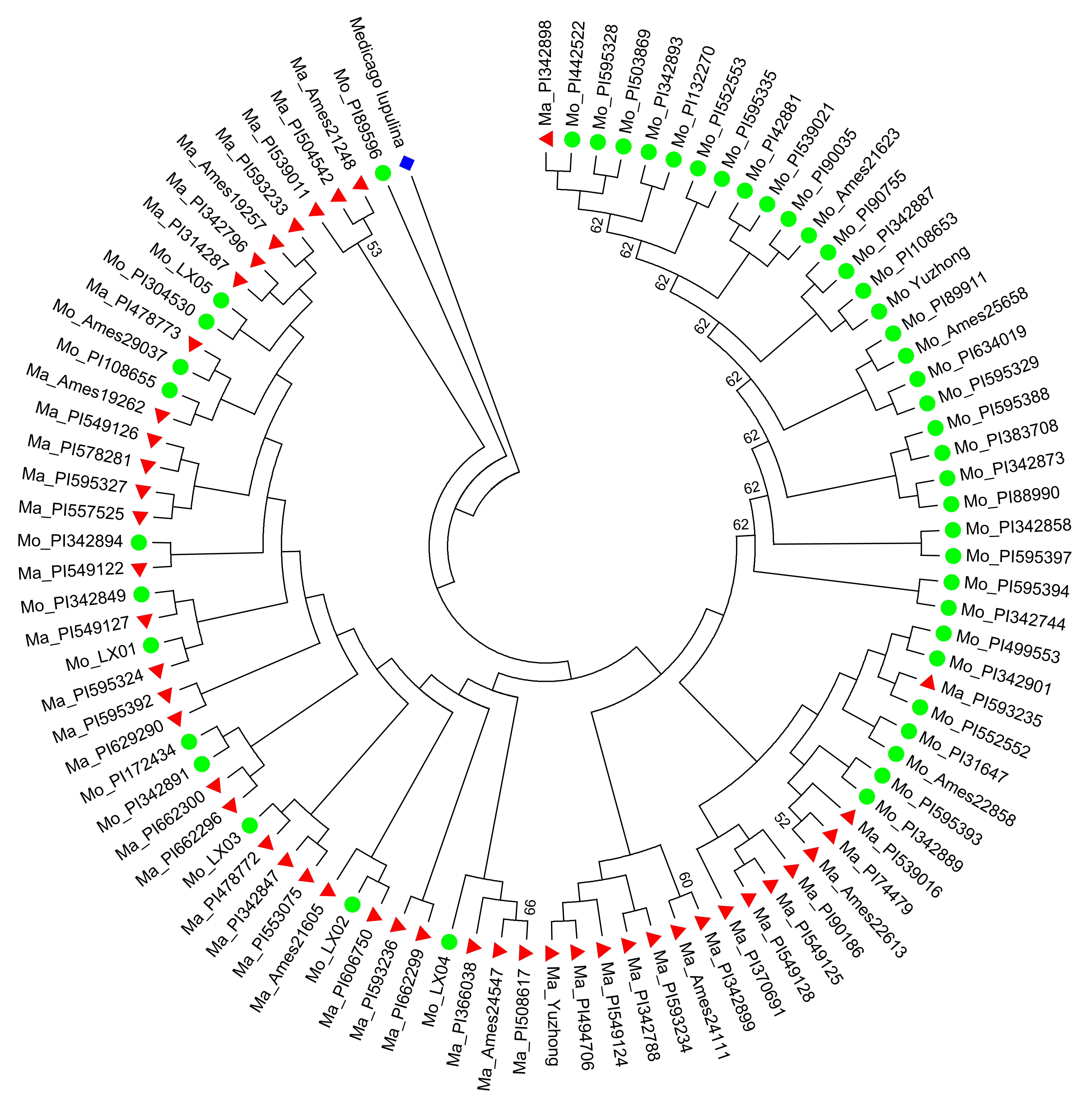

Supplement: Supplementary file 1 [file molecules-23-00810-s001.zip › Supplementary files/Figure S2.tif]

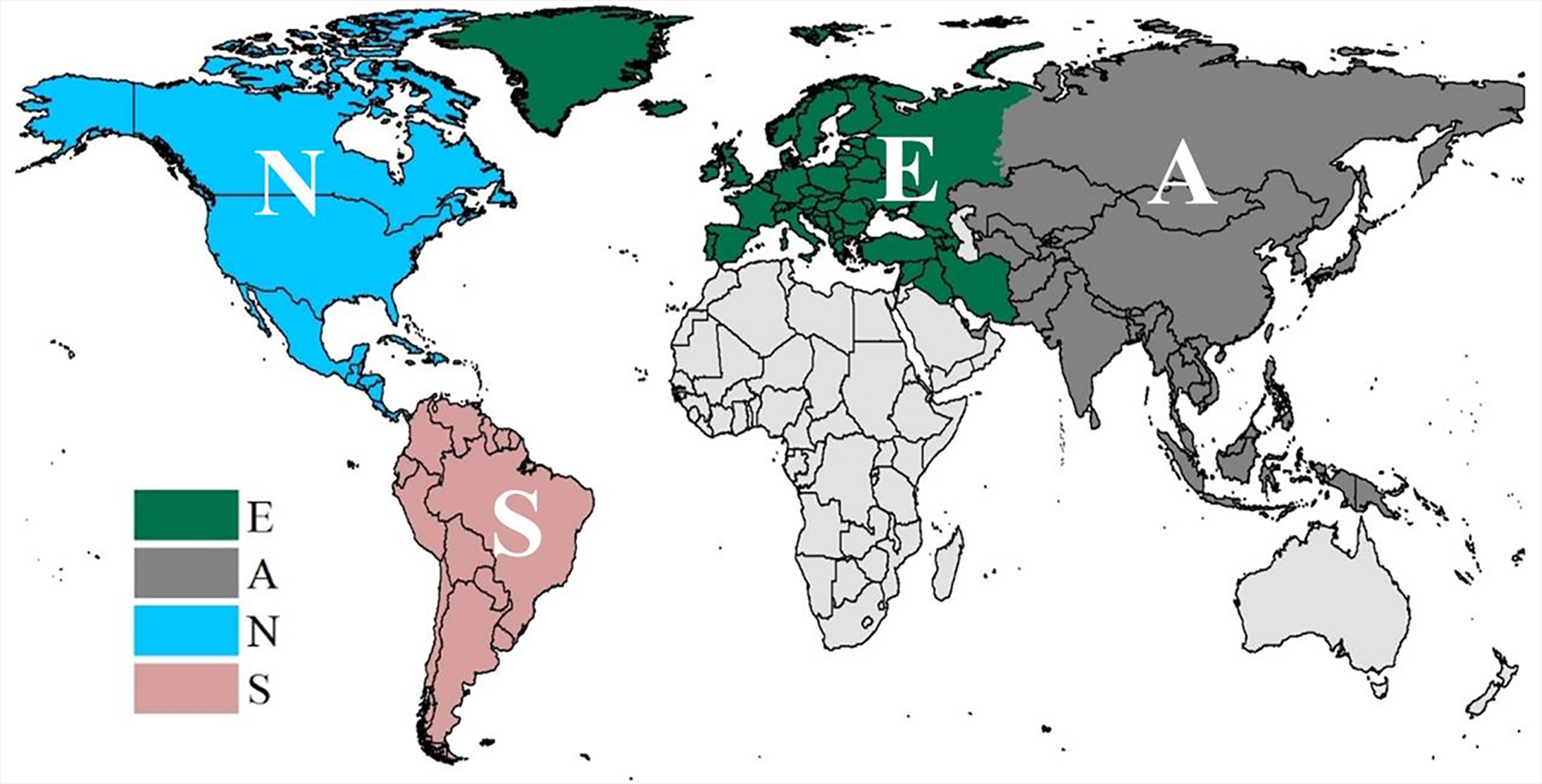

Supplement: Supplementary file 1 [file molecules-23-00810-s001.zip › Supplementary files/Figure S3.tif]
